# Safe focused ultrasound-mediated blood-brain barrier opening is driven primarily by transient reorganization of tight junctions

**DOI:** 10.21203/rs.3.rs-6701853/v1

**Published:** 2025-05-30

**Authors:** Elisa Konofagou, Rebecca Noel, Tara Kugelman, Maria Karakatsani, Sanjid Shahriar, Moshe Willner, Claire Choi, Yusuke Niimi, Robin Ji, Dritan Agalliu

**Affiliations:** Columbia University

## Abstract

Focused ultrasound (FUS) with microbubbles opens the blood-brain barrier (BBB) for targeted drug delivery into the brain. How brain endothelial cells (BECs) respond to either low acoustic pressures known to open the BBB transiently, or high pressures that cause brain damage, is incompletely characterized. Here, we apply FUS at low (450 kPa) and high (750 kPa) pressures in mice where BBB tight junctions are labelled with eGFP and find that arteriole and capillary BECs respond to low pressure by a transient reorganization of tight junctions associated with BBB opening. Moreover, this process does not depend on caveolae. In contrast, BBB opening at high pressure is associated with tight junction obliteration even after 72 hours, allowing persistent fibrinogen passage and microglial activation. Single-cell RNA-sequencing of BECs from FUS-BBBO mice shows that the transcriptomic responses of BECs exposed to high pressure are dominated by the stress response and cell junction disassembly, whereas lower pressure induces primarily genes responsible for intracellular repair. Therefore, transient reorganization of tight junctions and repair responses mediate safe BBB opening for therapeutic delivery.

## INTRODUCTION

Targeted delivery of therapeutics to the central nervous system (CNS) to treat neurological and psychiatric diseases remains a challenge due to the presence of the blood-brain barrier (BBB) formed by high resistance tight junctions (TJ) between brain endothelial cells (BECs) with a low rate of caveolar-mediated transport ^3-5^ and specialized transporters.^1,2^ Over the past two decades, neurosurgical, pharmacological and physiological strategies have been developed to circumvent the BBB. Focused ultrasound (FUS) with systemically delivered microbubbles (< 10μm in diameter) is a technology that increases BBB permeability locally, reversibly and non-invasively to facilitate targeted CNS drug delivery.^6^ At low acoustic pressures, the cavitating microbubbles undergo stable volumetric oscillations (stable cavitation), whereas at high acoustic pressures the microbubbles may experience inertial cavitation.^7-11^ Multiple studies have optimized the frequency,^12^ acoustic pressure,^13-15^ pulse duration,^16,17^ pulse and burst repetition frequency^18^ and microbubble size^19,20^ used to open the BBB in a safe and effective manner in rodents, non-human primates^21,22^ and humans.^23,24^ Using this technology, numerous chemotherapeutic agents,^25-27^ antibodies,^28,29^ neurotrophic factors,^30,31^ adeno-associated viruses, ^32,33^ and neural stem-cells^34^ have been delivered across the BBB in many neurological diseases.

Although parameters for safe effective BBB opening in rodents, non-human primates, and humans have been worked out, the molecular mechanisms mediating FUS-induced BBB opening (FUS-BBBO) and the transcriptomic response of BECs at low or high acoustic pressures remain incompletely characterized. Previous transmission or immunoelectron microscopy (EM) studies have found that FUS with a high acoustic pressure (1MPa), 1.5 MHz or 1.63 MHz center frequency transducer, and 100 ms pulse duration increases the number of BEC caveolae leading to formation of trans-endothelial channels to transport therapeutics across the BBB.^35^ High acoustic pressures (1 MPa) and shorter pulse durations (10 ms) with the same 1.5 MHz center frequency transducer also induce loss of BBB TJ proteins by immune EM within one hour after FUS, suggesting a potential increase in paracellular BBB permeability.^36^ In contrast, at pressures ranging from 200 to 800 kPa using a 1.029 MHz and 1.2 MHz center frequency transducer, two-photon imaging studies have demonstrated a slow diffusion found primarily in larger caliber vessels comparable to transcellular transport, followed by a quick diffusion in smaller caliber vessels comparable to paracellular diffusion likely through disrupted TJs.^37,38^ Thus, FUS can change both transcellular and paracellular BBB permeability depending on the pressure.

Here, we examined endothelial TJ morphology in CNS blood vessels of *Tg::eGFP-Claudin5* transgenic mice where TJs are labelled with eGFP^5,39^ at two distinct pressures and time points to correlate temporal changes in FUS-BBBO with TJ structural alterations. We have chosen a 450 kPa pressure associated with safe and reversible BBB opening, and a 750 kPa one known to cause irreversible damage.^40,41^ We have selected two time-points: a) 1 hour post-FUS, when the BBB permeability is increased at both pressures, and b) 72 hours post-FUS, when the BBB is resealed at low pressure, but remains open at high pressure.^40,41^ We report that FUS-BBBO is associated with permanent damage of BBB TJs in arterioles and capillaries by confocal microscopy within 1 hour only at high pressure; these junctions remain unrepaired even at 72 hours. This allows persistent fibrinogen passage at sites of BBB leakage and microglial activation. In contrast, at low safe pressures BBB opening occurs due to transient TJ reorganizations in arterioles and capillaries in both wild-type and *Cav1^−/−^* mice, suggesting a caveolar-independent mechanism. Single-cell RNA sequencing reveals that transcriptome responses of BECs exposed to high FUS pressure at both 1 and 72 hours are dominated by genes belonging to the stress response and junction protein disassembly, whereas lower pressures primarily drive genes responsible for intracellular repair. Thus, low pressures can safely and transiently open the BBB to deliver therapeutics to the brain primarily through transient reorganization of TJs.

## RESULTS

### FUS increases BBB permeability locally in Tg::eGFP-Claudin5^+/−^ mice

**Figure 1:**
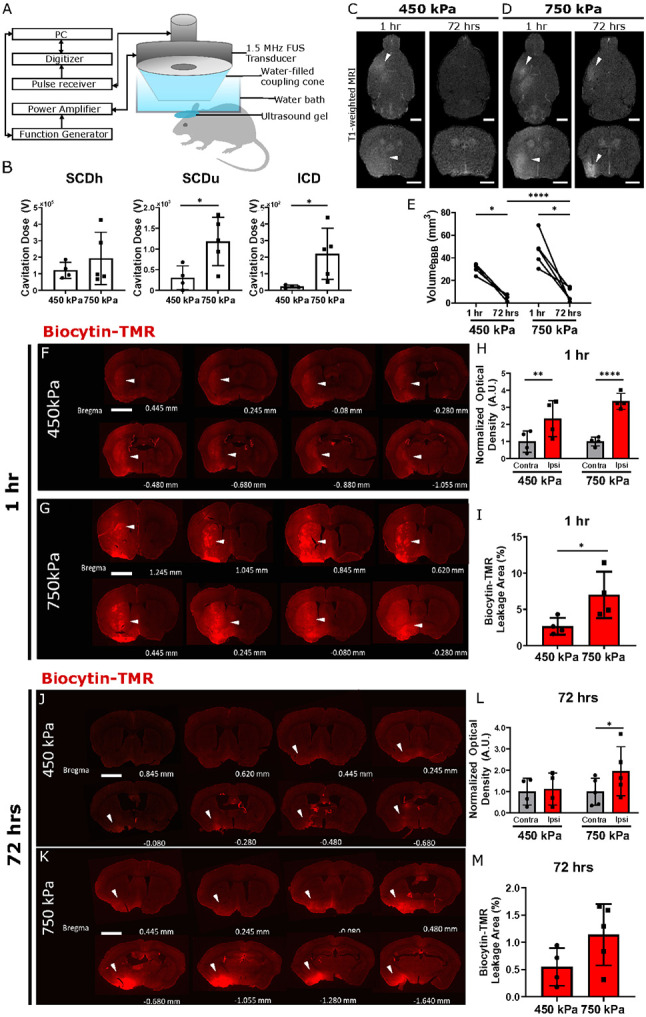
FUS with microbubble induces dynamic transient BBB opening only at safe pressures. (**A**) Schematic diagram showing a 1.5 MHz focused ultrasound transducer targeting the caudate/putamen region of the brain through the scalp and skull. (**B**) Quantification of the stable cavitation dose (SCD) for harmonics (SCDh) and ultra-harmonics (SCDu), along with the inertial cavitation dose (ICD) for 450 kPa (N=4 mice) and 750 kPa (N=5 mice). Signals were collected from a passive cavitation detector (PCD) during a 120 second interval of sonication. (**C-D**) Axial and coronal evaluation of BBB opening at 45 min and 71 hours and 45 min post FUS with contrast-enhanced T1-weighted MRI in the caudate / putamen region for 450 kPa (**C**) and 750 kPa (**D**) pressures. The BBB becomes permeable at both pressures after 1 hour; however, the BBB is permeable only at 750 kPa after 72 hours. (**E**) Quantification of the BBB leakage volume at 45 min following gadolinium injection administered immediately and 71 hours post FUS (N=4 mice/group; *p<0.05 and ****p<0.0001; two-way ANOVA with Sidak’s multiple comparison test). (**F, G**) *Tg::eGFP-Claudin5^+/−^* mice were injected with biocytin-TMR (870 Da) tracer after 1.5 Mhz frequency FUS to determine BBB permeability. Brain sections from *Tg::eGFP-Claudin5*^+/−^ mice showing biocytin-TMR tracer leakage from the vasculature on the ipsilateral caudate/putamen region 1 hour following FUS-mediated BBB opening at both 450 kPa and 750 kPa. (**H**) Quantification of biocytin-TMR fluorescence intensity normalized to optical density between ipsilateral and contralateral matched regions of interest (ROIs) at 450 kPa and 750 kPa pressures 1 hour after FUS (N=4 mice/group; **p<0.01 and ****p<0.0001; two-way ANOVA with Sidak’s multiple comparison test; a.u., arbitrary unit). (**I**) Quantification of biocytin-TMR leakage area at 450 kPa and 750 kPa one hour after FUS (N=4 mice/ group; *p<0.05; Student’s t-test). (**J-K**) Brain sections from *Tg::eGFP-Claudin5^+/−^* mice showing biocytin-TMR tracer leakage in the caudate/putamen region 72 hours following FUS-mediated BBB opening at 450 kPa (N=4) and 750 kPa (N=5). There is no biocytin-TMR in the brains of mice that received FUS at low pressure (450 kPa), except choroid plexus. (**L**) Quantification of biocytin-TMR fluorescence intensity normalized to optical density between ipsilateral and contralateral matched ROIs at 450 kPa (N=4) and 750 kPa (N=5) 72 hours after FUS-mediated BBB opening (*p<0.05; two-way ANOVA with Sidak’s multiple comparison test; a.u., arbitrary unit). (**M**) Quantification of biocytin-TMR leakage area at 450 kPa (N=4) and 750 kPa (N=5) 72 hours after FUS. Student’s t-test was used for statistical comparisons (p=0.1106). All data are presented as mean± standard deviation. Arrowheads for Figure 1F, G, J and K denote the area of biocytin extravasation. See also **Extended Data Figure 1**.

To analyze FUS-mediated temporal changes in BBB permeability and TJ morphology by confocal microscopy, we targeted non-invasively a focused ultrasound beam to the left caudate and putamen regions of *Tg::eGFP-Claudin5^+/−^* mice.^5,39^ Immediately preceding sonication, mice were injected intravenously with microbubbles and biocytin-TMR, a small (~ 890 Da) fluorescent tracer to assess BBB permeability.^5,39,42,43^ FUS-BBBO was performed at low^40,44^ (450 kPa) and high^40,41^ (750) pressures (Fig. 1A). Passive cavitation detection signals were collected and cumulative stable and inertial cavitation doses were calculated for each pressure (**Extended Data** Fig. 1). Harmonic acoustic signals associated with stable cavitation^7^ were detected at both pressures (**Extended Data** Fig. 1 A, B). At high pressure (750 kPa), ultra-harmonics^45^ and broadband emissions were also detected (**Extended Data** Fig. 1B, D, F), indicative of inertial cavitation.^7^ The stable cavitation dose for harmonics was not different between 450 and 750 kPa (Fig. 1B).

BBB leakage was initially assessed with contrast enhanced T1-weighted magnetic resonance imaging (MRI) after injection of Gd-DPTA-BMA (~ 540 Da) immediately or 71 hours post-FUS. Contrast enhancement (i.e., diffusion of Gd-DPTA-BMA across the BBB) was significantly greater at 1 compared to 72 hours for both pressures, and at 750 kPa compared to 450 kPa by 72 hours (Fig. 1C-E). Signal enhancement was significantly greater in mice exposed to high compared to low pressure at 72 hours, suggesting restored BBB function at low pressure and sustained BBB damage at high pressure. Similarly, diffusion analysis of a fluorescent tracer (biocytin-TMR; 890 Da) across the BBB into the brain parenchyma showed a 2- and 3.5-fold higher normalized optical density in the ipsilateral compared to the contralateral hemisphere in tissue sections 1 hour after FUS at low and high pressures, respectively (Fig. 1F-H). The area of biocytin-TMR leakage was 4-fold higher in mice sonicated at 750 kPa compared to 450 kPa within 1 hour after FUS (Fig. 1I). By 72 hours there was no difference between ipsilateral and contralateral hemispheres at 450 kPa, suggesting effective BBB closure (Fig. 1J, L); however, the biocytin-TMR leakage intensity was 2-fold greater on the ipsilateral hemisphere at 750 kPa, indicating sustained BBB disruption (Fig. 1K, L). The area of biocytin-TMR leakage at 72 hours after FUS was higher in mice exposed to 750 kPa compared to 450 kPa pressures (Fig. 1M), in contrast to the Gd-DPTA-BMA permeability (Fig. 1E). Overall, these findings are consistent with our prior studies showing higher BBB permeability at 72 hours in high compared to low FUS pressure. Moreover, the BBB opens transiently only at low (safe) acoustic pressures but remains permeable at higher (damaging) pressures over time.

### Loss of tight junctions in arterioles and capillaries contributes to persistent FUS-mediated BBB opening at high acoustic pressures

**Figure 2:**
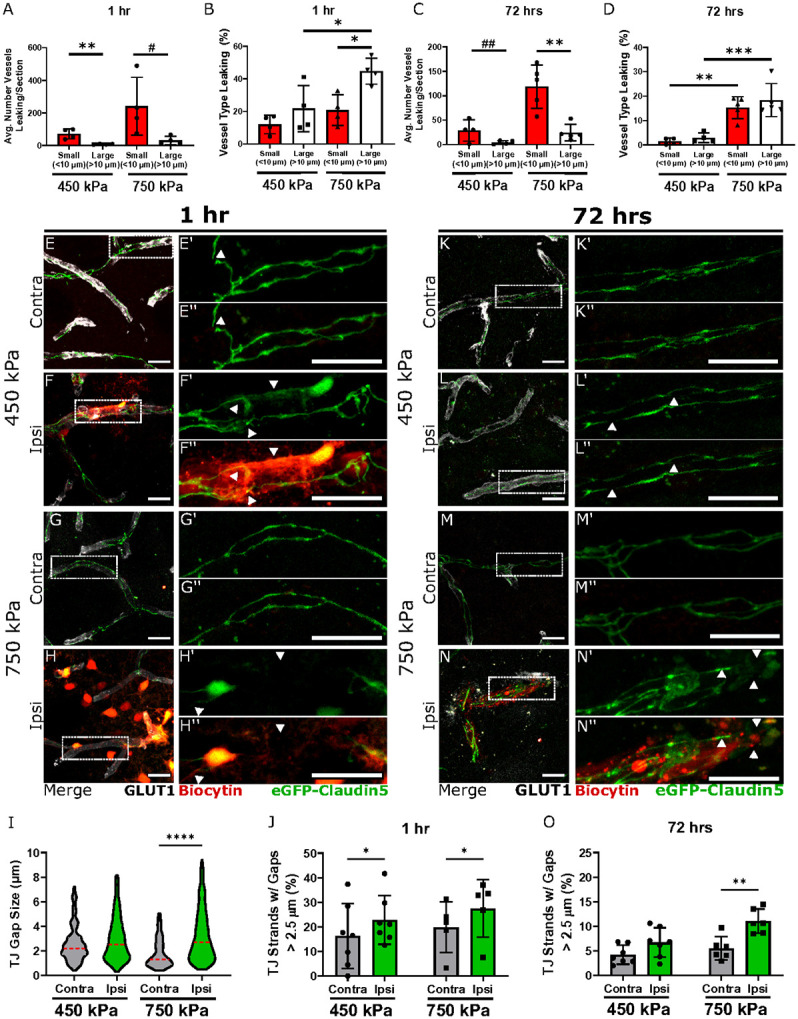
Persistent structural abnormalities in capillary tight junctions visualized by confocal microscopy correlate with continuous BBB opening at unsafe pressures. (**A**) Quantification of the average number of leaky small (<10 μm) and large (>10μm) vessels per brain section at 450 kPa and 750 kPa 1 hour following 1.5 MHz FUS sonication (N=4 mice / group; **p<0.01; #p = 0.0569; Student’s t-test). (**B**) Quantification of the fraction of small and large leaky vessels at 450 kPa and 750 kPa one hour after FUS sonication. The fraction of leaky vessels is higher for larger, than smaller, vessels at high pressure and there are significantly more large leaky vessels at high than low pressure (N=4 mice/group; *p<0.05; two-way ANOVA with Sidak’s multiple comparison test). (**C**) Quantification of the average number of leaky small (<10 μm) and large (>10μm) vessels per brain section at 72 hours following 1.5 MHz FUS sonication at 450 kPa and 750 kPa (N=4 mice / group; **p<0.01, ##p = 0.0682; Student’s t-test). (**D**) Quantification of the fraction of small and large leaky vessels at 750 kPa compared to 450 kPa at 72 hours after FUS sonication (N=4 mice/group; ***p<0.001, **p<0.01 two-way ANOVA with Sidak’s multiple comparison test). (**E-H”; K-N”**) Maximum intensity projection images of 25μm-thick caudate putamen sections from *Tg::eGFP-Claudin5*^*+/−*^ mice sonicated at 450 (**E-F”**) and 750 kPa (**G-H”**). The images show capillaries within the ipsilateral and contralateral caudate / putamen regions 1 (**E-H”**) and 72 (**K-N”**) hours after FUS sonication. eGFP (green) labels TJs between endothelial cells. Biocytin-TMR tracer leakage from blood vessels (red) is seen exclusively in the parenchyma of the treated hemisphere. GLUT-1 (white) labels endothelial cells. The absence of TJ strand in vessels is indicated by white arrowheads. (**I-J**) Quantification of the fraction of TJ strands with gaps between 0.4-2.5 μm (**I**) and > 2.5 μm (**J**) at 450 kPa and 750 kPa. The fraction of TJ strands with gaps > 2.5 μm is significantly different in the ipsilateral compared to the contralateral hemisphere at 750 kPa (N=4 mice / group; *p < 0.05; one-way ANOVA with Sidak’s multiple comparison test). (**O-P**) Quantification of the fraction of TJ strands with gaps between 0.4-2.5 μm (**O**) and > 2.5 μm (**P**) at 450 kPa and 750 kPa. The fraction of TJ strands with gaps greater than 2.5 μm is significantly different in the ipsilateral compared to the contralateral hemisphere at 750 kPa (N=5 mice / group; *p < 0.05; one-way ANOVA with Sidak’s multiple comparison test). All data are presented as mean± standard deviation. Scale bars = 20 μm. See also **Extended Data Figures 2-7**.

To correlate changes in FUS-BBBO at both pressures with TJ structural alterations, we analyzed the number and type of leaky vessels with structural TJ abnormalities via immunofluorescence and confocal imaging in the caudate / putamen regions at 1 and 72 hours post-FUS. The majority (~ 90%) of Glut1 + leaky vessels were small diameter (< 10 μm) vessels for both pressures at 1 hour after FUS (Fig. 2A). There was a 3-fold increase in the number of leaky vessels with small diameter and a 4-fold increase in the number of those with large diameter (> 10 μm) at high compared to low pressures after 1 hour (Fig. 2A). In contrast, a greater percentage of large diameter vessels were significantly leaky at 750 kPa compared to 450 kPa (Fig. 2B). Thus, higher pressures increase the probability of opening larger diameter vessels. The larger leaky vessels were exclusively arterioles based on TJ morphology^5,39^ and expression of **α**-smooth muscle actin (**α**-SMA; data not shown). We could not detect tracer leakage from venules or larger veins (data not shown). By 72 hours, the average number of leaky vessels at both pressures was lower than 1 hour post-FUS, and the number of leaky small vessels was higher than large ones (Fig. 2C). However, the vessel diameter was a poor predictor of the area and intensity of tracer leakage at either pressure or time after FUS-BBBO (Extended Data Fig. 2). At 72 hours after FUS-BBBO, there was no significant difference between the fraction of leaky small vs. large diameter vessels in each pressure; however, the overall percentage of leaky vessels was greater at high (750 kPa) compared to low (450 kPa) pressure (Fig. 2D), consistent with biocytin-TMR leakage across the BBB (Fig. 1J-M). Thus, low safe pressures do not preferentially disrupt smaller diameter vessels and this is largely resolved by 72 hours. In contrast, large vessels are preferentially disrupted at high pressures, and both vessels remain leaky 72 hours after FUS.

We analyzed the morphology of eGFP^+^ TJs in capillaries of *Tg::eGFP-Claudin5^+/−^* mice using confocal microscopy 1 and 72 hours after FUS for both pressures and quantified the fraction of capillary TJs containing “pathological” gaps larger than 2.5 μm in both ipsilateral and contralateral caudate and putamen regions as described ^5,39,43^. There was a 1.5-fold increase in the fraction of TJ strands with gaps > 2.5 μm in leaky capillaries for both pressures at 1 hour after FUS, although the lower pressure had a higher variability (Fig. 2E-H”, 2I, J; Extended Data Fig. 3). Moreover, a large number of leaky capillaries lacked completely TJ strands at 750 kPa, whereas these were absent at 450 kPa (Fig. 2H’, F’). By 72 hours, although there was no difference in the fraction of capillary TJ with gaps > 2.5 μm between the ipsilateral and contralateral regions at 450 kPa (Fig. 2K-L”, O), there was still a significant fraction of capillary TJ with gaps > 2.5 μm in the ipsilateral hemisphere at 750 kPa (Fig. 2M-N’, O). Thus, high acoustic pressures induce persistent structural damage of capillary TJs by confocal microscopy.

Arterioles constitute a small percentage of leaky vessels at both 1 and 72 hours (Fig. 2A-D); however, a compromised BBB at the arteriole level can cause significant damage to the brain. The percentage of TJ strands with big gaps was 4-fold higher in leaky arterioles of the ipsilateral striatum at high pressures 1 hour after FUS-BBBO (Extended Data Fig. 4A-D”, I, J) with no significant statistical difference at low pressure (Extended Data Fig. 4A-B”, I, J). Thus, high acoustic pressures induce more acute structural damage to TJs in arterioles than capillaries. By 72 hours the fraction of TJs with gaps was 6-fold higher in the arterioles of the ipsilateral hemisphere with high pressure FUS-BBBO (Extended Data Fig. 4E-F”, I, K). However, we did not find similar abnormalities with ZO-1 in Glut1 + vessels exposed to 450 and 750 kPa at 72 hours after FUS (Extended Data Fig. 5). In summary, transient BBB opening seen at low pressures correlates with transient loss and repair of TJ strands. Conversely, high acoustic pressures induce persistent structural damage to TJs in both capillaries and arterioles that cause long-term BBB damage.

Fibrinogen is a plasma protein involved in coagulation, inflammation and tissue repair that does not normally cross the BBB.^49^ Following BBB disruption in neuroinflammation or neurodegeneration, fibrinogen activates microglia initiating neuropathological deficits.^50-54^ One hour following the FUS sonication, the area of fibrinogen leakage was higher on the ipsilateral compared to the contralateral regions at both pressures (Extended Data Fig. 6A-E). Fibrinogen leakage was present only near vessels with biocytin-TMR leakage where there was ameboid microglia (Extended Data Fig. 6B”, D”). By 72 hours, we could not detect any fibrinogen at any pressure (Extended data Fig. 6F-I, J) indicative of BBB repair. 3D texture-based volume renderings of 25 μm Z stack confocal images allowed a visualization of microglia and biocytin at each timepoint and pressure (Extended Data Movie S1–4).

Caveolae-mediated transcytosis ^3-5^ is a key feature of the BBB, prompting us to evaluate whether upregulation in caveolar-mediated transcellular transport could account for the transient BBB opening at low pressure. We compared BBB permeability after FUS between *Tg::eGFP-Claudin5^+/−^* mice and *Tg::eGFP-Claudin5^+/−^*; *Cav1^−/−^* lacking caveolae. There was no difference in BBB opening volume assessed by MRI, and the area or intensity of biocytin-TMR leakage in the caudate and putamen regions was similar between the two genotypes (Extended Data Fig. 7). Thus, transient BBB opening at safe pressures is likely not mediated by upregulation in caveolar-mediated transport in BECs, but by transient TJ reorganization.

### Single cell RNA-sequencing reveals FUS-induced transcriptional changes related to injury and repair in BECs

**Figure 3:**
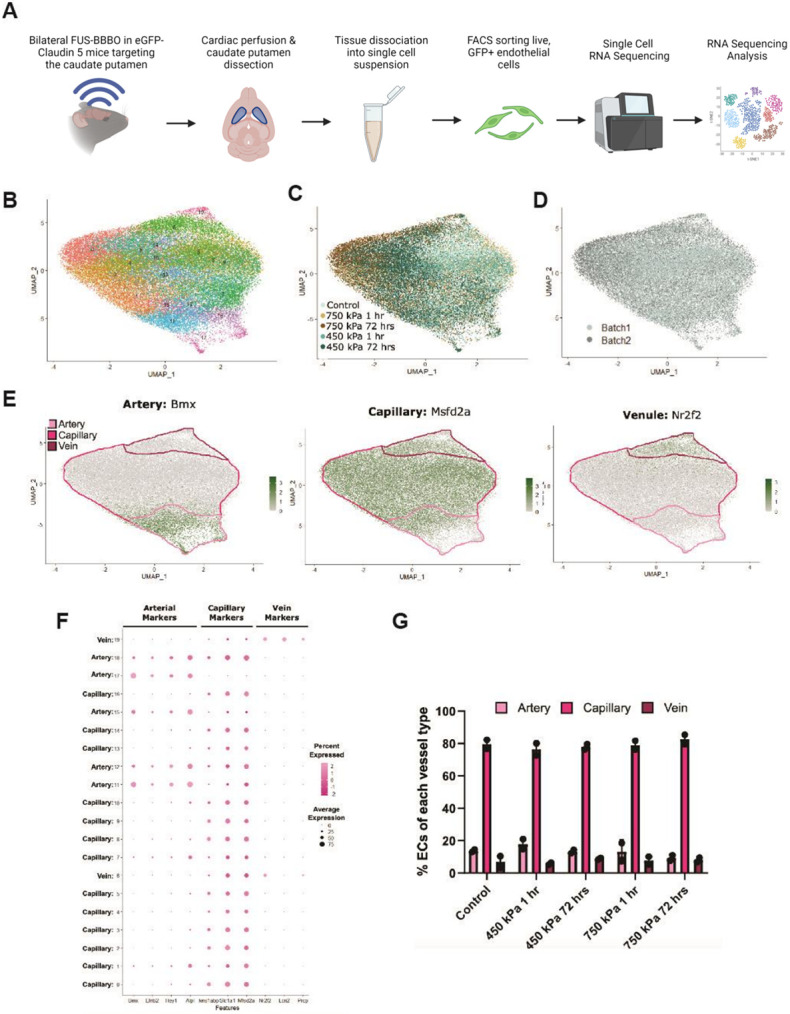
Single-cell RNA sequencing of endothelial cells after FUS and identification of EC subtypes. (**A**) Experimental workflow for single-cell RNA sequencing experiments of ECs after FUS. (**B-D**) Uniform manifold approximation and projection (UMAP) of ECs integrated and color coded by Seurat cluster, treatment group, and sequencing batch. (**E**) Clusters of arterial, capillary and venous cells identified by expression of canonical markers Bmx, Msfd2a and Nr2f2 respectively in the UMPA plot. (**F**) Dotted plot of canonical markers used to identify clusters to be classified as arterial, capillary and venous cells. The size of the dot indicates the number of cells and the color the intensity of expression. (**G**) Dotted bar plot showing the percentage of arterial, capillary, and venous ECs for each treatment group. See also **Tables S1-S4**.

To determine the transcriptomic response of BECs to low and high pressures at 1 and 72 hours after FUS-BBBO, we dissociated the caudate and putamen regions from *Tg::eGFP-Claudin5^+/−^* brains after bilateral FUS into single cells, FACS-sorted eGFP^+^ BECs and performed single-cell RNA-sequencing (scRNAseq) and analysis^55^ (Fig. 3A, **Table S1**). After quality filtering^56^ and *in silico* BEC selection based on defined markers [*Pecam1, Cldn5,* and *Erg*],^57^ we performed graph-based clustering to separate BECs sequenced from 10 brain samples (2 samples per condition and 2 healthy controls; **Table S1**) into distinct clusters based on transcriptome expression profiles using high resolution. This approach separated BECs into 20 clusters visualized using a Uniform Manifold Approximation and Projection (UMAP) representation (Fig. 3B-D). This approach avoids pitfalls arising from incorrect clustering boundaries using lower resolution.^58^ We assigned a subtype identity to each BEC cluster based on expression of canonical markers for arteries, capillaries and venules (Fig. 3E, F). Expression of arterial and venous markers peaked at opposite ends of the UMAP plot separated by capillary clusters (Fig. 3E, F). The second largest variation axis was the FUS-BBBO pressure at 72 hours versus control conditions (healthy BECs). Capillary BECs were the largest population, arterioles BECs comprised the second one, and venule BECs were the smallest population for each condition (Fig. 3G).

**Figure 4:**
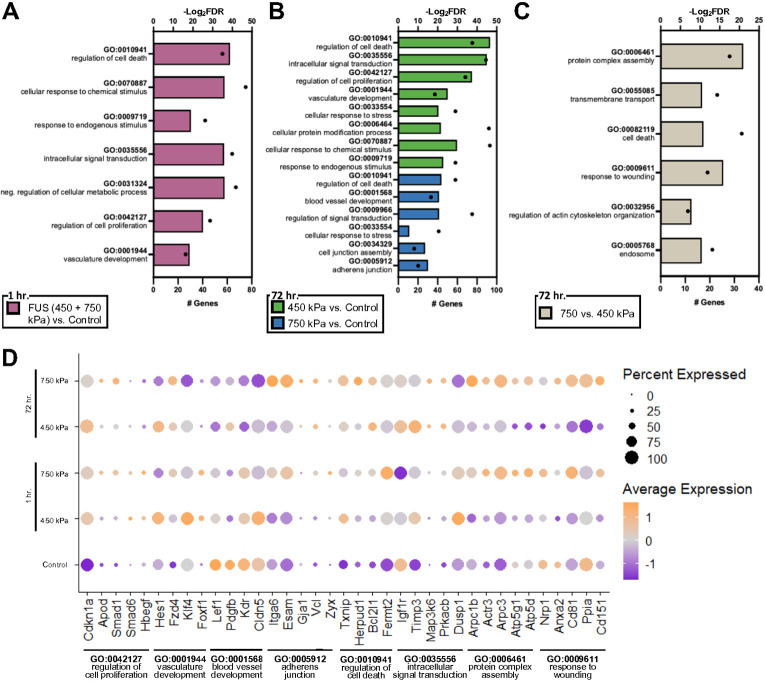
Gene ontology analysis of differentially expressed genes in capillary BECs for each condition from single cell RNA sequencing. (**A**) Gene ontology terms upregulated in FUS (450 + 750 kPa) capillary cells compared to untreated controls 1 hour post-FUS-BBBO. (**B**) Gene ontology terms upregulated in capillary cells exposed to 450 kPa (green) and 750 kPa (blue) compared to untreated controls, 72 hours post-FUS-BBBO. (**C**) Gene ontology terms upregulated in capillary cells exposed to 750 kPa compared to 450 kPa cells 72 hours post-FUS-BBBO. In all bar plots the bar represents the −Log_2_(FDR) (top axis), and the dot represents the number of significantly annotated genes (bottom axis). (**D**) Dot plot showing relative expression of key genes annotated to the upregulated gene ontology terms for each treatment group and untreated controls. The size of the dot indicates the number of cells and the color the intensity of expression. See also **Extended Data Figure 8** and **Tables S5-S7**.

To identify key transcriptome signatures differentiating BECs from each condition, we performed differential gene expression analysis comparing FUS-BBBO 1 hour (the transcriptome responses of capillary ECs to 450 and 750 kPa 1 hour post-FUS were statistically indistinguishable, so they were grouped together), 450 kPa 72 hours, or 750kPa 72 hours to control-enriched BEC clusters for each vessel subtype (**Tables S2-S4**). This data was used to conduct gene ontology (GO) analysis in DAVID and gene set enrichment analysis (GSEA) using curated and comprehensive gene catalogs for various cellular and molecular pathways and functions from the Molecular Signatures Database v6.1 (*MSigDB:*software.broadinstitute.org/ gsea/msigdb/genesets.jsp),^59,60^ and determine which biological processes were significantly up- and downregulated for each condition (Tables S5-S8). The transcriptome analysis of capillary BECs one hour-post-FUS revealed upregulation of several genes and GO terms related to “cell death (e.g. *Txnip*)”, “intracellular signal transduction (e.g. *Hes1, Smad1, Smad6*),” “regulation of cell proliferation (e.g. *Cdhkn1a*),” and “vasculature development (e.g. *Klf4, Hes1*)” relative to untreated controls (Fig. 4A, D; Tables S2, S5). However, the acute capillary BECs response to FUS was more complex as other transcripts related to “vascular development” (*e.g. Pdgfb, Kdr, Lef1, Nrp1*) were downregulated compared to control BECs (Fig. 4A, D, Tables S2, S5). This complex transcriptional BEC response suggests initiation of a potential repair response in additional to the injury response at the acute phase (1 hour) post-FUS-BBBO.

GSEA analysis of capillary BECs revealed upregulation of “inflammation”, “Notch signaling” and “endothelial to mesenchymal transition” at 1 hour post-FUS (Extended Data Fig. 8A). A similar transcriptional response was observed also in arterioles and venules 1 hour post-FUS. Arterioles exposed to 750 kPa showed increased expression of transcripts annotated to “regulation of cellular component movement” and “cell-substrate junction” pathways, and venules exposed to 450 kPa demonstrated upregulation of “blood vessel development” and “regulation of cellular component movement” (Extended Data Fig. 8C, D, Table S2, S6, S7). Therefore, all three BEC subtypes respond dynamically at the acute phase (1 hour) post FUS to upregulate transcripts related to injury (e.g. “cell death”, “inflammation”) and repair (e.g. “cell proliferation”, “cell migration”, “vascular development”) processes regardless of the applied pressure.

The GO term analysis of DEGs upregulated in capillary BECs at 72 hours post – FUS at 450 kPa compared to control BECs revealed upregulation of pathways such as “regulation of cell death” (e.g. *Txnip, Bcl21b*), “intracellular signal transduction,” “regulation of cell proliferation” (e.g. *Cdnk1a*), “vascular development” (*Notch1, Notch4, Hes1, Klf4, Clic4*) and “cellular response to stress” (e.g. *Slc2a1, Slc38a2*) (Fig. 4B, D, Table S3, S5). Surprisingly, GSEA analysis of DEGs at 72 hours versus 1 hour post-450 kPa or control ECs showed downregulated pathways for “blood-brain barrier”, “cell proliferation”, “angiogenesis”, “EC migration”, “tip cells”, “Wnt/β-catenin, Notch and TGF-β signaling” (**Extended Data Fig. 8A, B, Table S8**). These data indicate that BECs are undergoing vascular growth and BBB maturation at 72 hours post-FUS at low pressures.

Similarly, the GO term analysis of DEGs upregulated in capillary BECs at 72 hours post-FUS at 750 kPa showed also upregulation of pathways such as “regulation of cell death” (e.g. *Txnip, Bcl21b*), “intracellular signal transduction,” “regulation of cell proliferation” (e.g. *Cdnk1a*), and “vascular development” (*e.g. Notch1, Notch4, Hes1, Klf4, Clic4*) compared to control ECs (Fig. 4B, D). In contrast to 450 kPa, GSEA analysis revealed that processes such as “blood-brain barrier”, “inflammation”, “tip cells” were upregulated at 72 hour post-750 kPa, whereas those to “angiogenesis”, “EC migration”, VEGF, TGF-β and non-canonical Wnt signaling” were downregulated compared to controls (**Extended Data Fig. 8A, B, Table S8**). Pathway analysis of upregulated DEGs in ECs after 72 hours after 750 kPa compared to 450 kPa revealed the presence of pathways related to “protein complex assembly,” (e.g. *Arpc1b, Arpc3, Actr3, Atp5d, Atp5g1*), “regulation of cell death,” and “response to wounding” (e.g. *CD81, CD151, Ppia*) (Fig. 4C, D). These transcriptome findings are consistent with the TJ analysis that BECs exposed to 450 kPa mostly recover by 72 hours post-FUS, whereas those exposed to 750 kPa are still undergoing cell death and early repair processes, since they were severely damaged by high pressure.

### Several major signaling pathways related to angiogenesis and barriergenesis change dynamically in BECs after FUS

**Figure 5:**
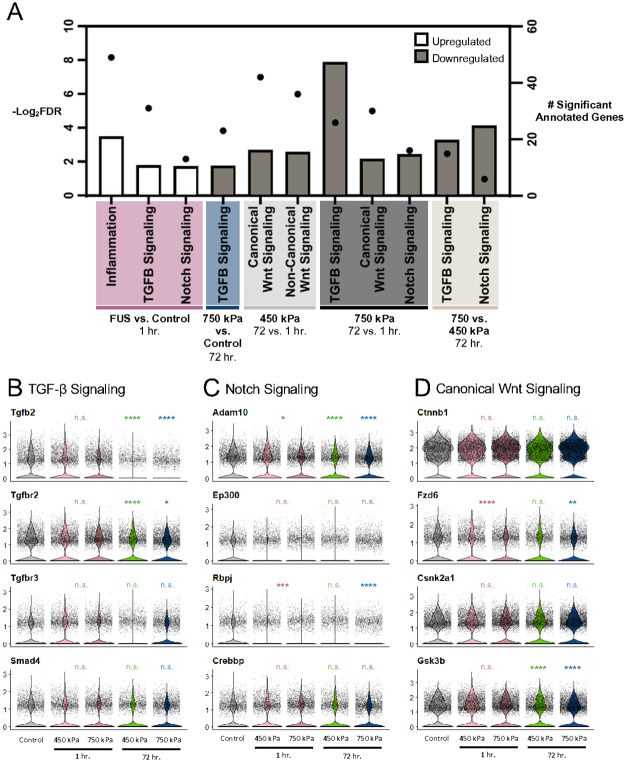
Gene set enrichment analysis (GSEA) results for capillary BECs. (**A**) GSEA terms up and downregulated in capillary cells for the indicated comparisons (P value cutoff < 0.3). Bars represent the −Log_2_(FDR) values (left y-axis), and dots represent the number of significant genes annotated to each term (right y-axis). Comparison label convention is “Measured vs. Reference”. Namely, “FUS vs Control” represents changes observed in FUS groups using the Control group as reference. (**B-D**) Violin plots showing the expression of representative genes annotated to TGF-β Signaling, Notch Signaling, and Canonical Wnt Signaling. Statistical significance was measured between each group and the Control using pairwise Wilcoxon rank sum tests to determine the adjusted p-value. FUS groups 1 hour post-FUS-BBBO were pooled for this analysis (450 + 750 kPa). Significance values are indicated as follows: * P ≤ 0.05, ** P ≤ 0.01 ***

In order to understand which signaling pathways critical for angiogenesis and barriergenesis change differentially in BECs after low and high FUS pressure, we performed a GSEA analysis for specific pathways. TGF-β signaling, which functions to inhibit cell proliferation, suppress inflammation and promote vascular development, is upregulated in capillary BECs one hour post-FUS consistent with increased inflammatory responses (Fig. 5A; Tables S5, S8). However, this pathway is largely downregulated by 72 hours relative to either control BECs, one hour post-FUS at 750 kPa, or 72 hours post-FUS 450 kPa (Fig. 5A; Tables S5, S8), consistent with reduced inflammation and initiation of angiogenesis. Several TGF-β signaling genes (e.g. Tgfb2 and Tgfbr2) were significantly downregulated in the 750 kPa group 72 hours post-FUS relative to other conditions (Figs. 5B).

Notch signaling, which is an important regulator of angiogenesis and BEC proliferation, changed dynamically after FUS. Notch signaling components (*e.g. Rbpj, Adam100)* were upregulated significantly in capillary BECs one hour post-FUS compared to control ECs (Figs. 5A; **Tables S5, S8**). However, the Notch pathway was largely downregulated by 72 hours post-FUS in capillary ECs, relative to those isolated from one hour post-FUS at 750 kPa, or 72 hours post-FUS at 450 kPa (Figs. 5D; Tables S5, S8). This was also consistent for arteriole BECs. In addition, arteriole BECs showed decreased VEGF signaling at 72 hours post 750 kPa relative to untreated controls (Fig. 5A). However, when comparing arterioles exposed to 750 kPa to those exposed to 450 kPa 72 hours post-FUS, GSEA analysis reveals increased expression of TGF-β and VEGF signaling. Therefore, several signaling pathways critical for angiogenesis and BEC proliferation change dynamically after FUS.

Wnt/β-catenin signaling is critical for CNS angiogenesis and BBB maturation, prompting us to examine its BEC transcriptional changes after FUS. GO and GSEA analyses did not show any significant differences in genes for Wnt/β-catenin signaling in either low or high pressure at the acute phase, compared to the untreated control group. However, canonical Wnt signaling was decreased at both pressure groups at 72 hours compared to one hour post-FUS (Fig. 5A, Tables S5, S8). Additionally, arterioles exposed to both pressures showed decreased expression of Wnt/β-catenin signaling at 72 compared to one hour post-FUS (Table S6). Overall, this analysis indicates a “return to normalization” at both pressures by 72 hours relative to one hour post-FUS, since Wnt/β-catenin signaling is reduced once angiogenesis and BBB maturation are complete.^1,2^

**Figure 6:**
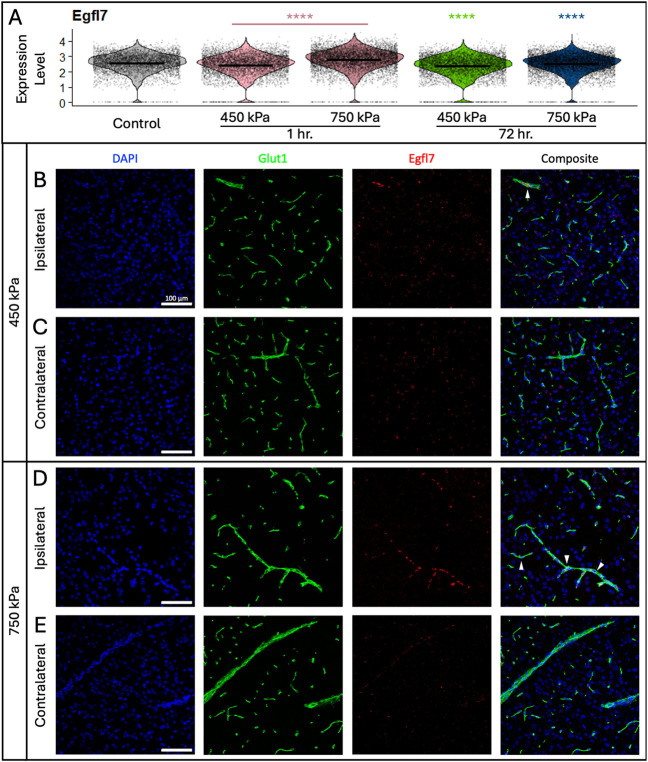
Glut1-positive BECs exposed to 750 kPa exhibit a sustained, FUS-induced increase in Egfl7 mRNA expression. (**A**) Violin plots showing expression of *Egfl7* mRNA across each of the treatment groups. Mean expression level is indicated by the bar in each plot. Statistically significant differences between each group and untreated controls are determined by Wilcoxon rank sum test, **** p ≤ 0.0001. BECs exposed to 450 kPa (**B**) exhibit modest increase in *Egfl7* mRNA expression compared to the untreated contralateral hemisphere (**C**). BECs exposed to 750 kPa (**D**) exhibit markedly increase in *Egfl7* mRNA levels relative to the untreated contralateral hemisphere (**E**). White arrowheads indicate Glut-1+ vessels with positive Egfl7 expression.

We validated some of the scRNAseq findings related to “angiogenesis” using fluorescent in situ hybridization (FISH) for two angiogenesis markers *Egfl7*and *Angpt2*^55^ with immunofluorescence for a vessel marker (Glut-1, Figs. 6, Extended Data Fig. 9). Violin plots from scRNA-seq data revealed significant increase for *Egfl7* and *Angpt2* mRNAs in the pooled 1 hour pressure group, and at 72 hours in both 450 and 750 kPa relative to untreated controls as determined by Wilcoxon rank sum test (Figs. 6A, Extended Data Fig. 9A). By FISH, *Egfl7* mRNA was slightly upregulated in Glut1-positive vessels exposed to 450 kPa on the ipsilateral hemispheres, whereas we could not detect any *Angpt2* mRNA upregulation (Fig. 6B, C and Extended Data Fig. 9B, C). In contrast both *Egfl7* and *Angpt2* mRNAs were highly upregulated in Glut-1 + vessels exposed to 750 kPa on the ipsilateral hemisphere (Fig. 6D, E and Extended Data Fig. 9D, E). These findings validate the scRNAseq data that “angiogenesis” is upregulated at 72 hours in both pressures conditions, with a more extreme and sustained response in the 750 kPa group. In conclusion, the BBB defects due to FUS promote an “angiogenic” program in CNS blood vessels to drive vascular repair.

## DISCUSSION

FUS with microbubbles allows non-invasive, localized, and transient increase in BBB permeability and presents a promising therapeutic method to deliver drugs into the brain. However, the cell biological mechanisms and transcriptome changes by which BECs respond acutely and sub-acutely to FUS-BBBO are not fully characterized. Here we show that BBB TJ structural integrity is compromised for a longer period (72 hours) at high, but not low, pressures in capillaries and to a lesser extent in arterioles. The diameter of cavitating microbubbles matches more closely to capillaries than arterioles, allowing a higher probability of physical interactions with their vessel walls.^72^ This may explain why the microbubbles are more efficient to disrupt capillaries TJs at both pressures. With increasing vessel diameter, there is increased microbubble expansion^73,74^ and inertial cavitation threshold,^75^ likely due to changes in the resonance frequency of the bubbles or additional constraints in the microvasculature.^76,77^ Therefore, microstreaming and shock waves events may occur more often in larger vessels,^7-11^ which is supported by our data that higher pressure disrupts TJs in larger than smaller vessels. Although we could not detect any venules that colocalized with the fluorescent tracer at either pressure, structural changes in BBB features have been reported in venules by immunoelectron microscopy at very high pressures (1 MPa), albeit at a lower percentage than capillaries and arterioles.^78^ Future EM studies will provide better insight whether there is vessel selectivity in BBB opening with low safe pressures.

TJs are stable in the healthy CNS but undergo dynamic remodeling in CNS diseases.^5,39,79,80^ Understable cavitation, we find significant disruption in both capillary and arteriole TJs segments with gapslarger than 2.5 μm within 1 hour post FUS at both pressures that likely underlie FUS-BBBO. However, by 72 hours, there is no difference in the fraction of TJ segments with larger gaps (> 2.5 μm) between the two hemispheres for 450 kPa, consistent with transient BBB opening. TJ protein degradation may occur as quickly as 20 minutes following sonication^38^ and TJ strands may be repaired within 1 hour post low pressure FUS-BBBO. Our transcriptome analysis of BECs within 1 hour post FUS revealed that “cellular responses to stress” and “junctional protein synthesis” are upregulated to cope with injury; however, this response has resolved back to baseline levels by 72 hours post-FUS. Stable cavitation may either change the turnover of TJ membrane proteins or stability of ZO proteins that anchor TJ transmembrane proteins to the cytoskeleton.^81^ Under high pressure (750 kPa) with inertial cavitation, capillaries exhibited a full absence of TJ strands, a phenomenon likely induced by shock waves generated by the collapse of microbubbles. These TJ deficits are not restored by 72 hours following sonication suggesting a permanent TJ damage. Transcriptome analysis of ECs exposed to 750 kPa confirm cell biological findings that sustained injury (e.g. “cell death”) and repair processes (e.g. “cell proliferation”, “cell migration”, “vascular development”) were observed at both acute and long-term timepoints at 750 kPa. Removal of complete TJ segments may not permit BECs to restore cell-cell junctions by 72 hours leading to longer-term structural and functional BBB impairment at high pressure.

It is possible that the transient opening of the BBB at low pressure are mediated by caveolae. However, we observed no differences in BBB opening between *Tg::eGFP-Claudin5^+/−^* mice and *Tg::eGFP-Claudin5^+/−^; Cav1*^*−/−*^ mice sonicated at 450 kPa, suggesting that caveolae do not play a significant role in the transient BBB opening at safe pressures after 1 hour. Increased number of caveolae have been reported in arterioles using lower center frequency transducer after FUS^78,82,83^ and caveolae-mediated endocytosis may be important for transporting large molecules across the BBB.^84^ Our data do not support a role for caveolar-mediated transport; however, transcriptomic response of BBB opening in *Cav1−/−* mice should be investigated in future studies.

The transcriptome analysis of BECs after FUS at both pressures revealed that arterioles, capillary and venules respond dynamically at the acute phase (1 hour) post FUS to upregulate transcripts related to injury processes (e.g. “cell death”, “inflammation”) and repair processes (e.g. “cell proliferation”, “cell migration”, “vascular development”) regardless of the applied pressure. Thus, ECs respond immediately to FUS injury to start repair processes. In contrast, there are distinct transcriptomic BEC responses between the two pressures by 72 hours. Capillary BECs upregulate pathways related to “cell death”, “intracellular signal transduction,” “regulation of cell proliferation”, “vascular development” and “cellular response to stress” by 72 hours at both pressures. Surprisingly, GSEA analysis with targeted showed downregulated pathways for “blood-brain barrier”, “cell proliferation”, “angiogenesis”, “EC migration”, “tip cells”, “Wnt/β-catenin, Notch and TGFβ signaling” in ECs from 450 kPa by 72 hours. In contrast to 450 kPa, GSEA analysis revealed that processes such as “blood-brain barrier”, “inflammation”, “tip cells” were upregulated at 72 hour post-750 kPa, whereas those to “angiogenesis”, “EC migration”, VEGF, TGFβ and non-canonical Wnt signaling” were downregulated compared to controls. Moreover, transcriptome comparison of BECs from 750 kPa and 450 kPa revealed the presence of pathways related to “protein complex assembly,” “regulation of cell death,” and “response to wounding”. BECs from 450 kPa are likely still undergoing vascular growth and BBB maturation at 72 hours post-FUS reflected in lower transcripts compared to controls. Moreover, our transcriptome findings are consistent with the concept that ECs exposed to 450 kPa mostly recover by 72 hours post-FUS, whereas those exposed to 750 kPa are still undergoing cell death and early repair processes, since they are damaged more extensively by high pressure.

The pathway analysis of BEC transcriptome after FUS revealed that two angiogenesis promoting pathways TGF-β and Notch signaling change dynamically after FUS. TGF-β signaling which functions to inhibit cell proliferation, suppress inflammation and promote vascular development, is upregulated in capillary BECs one hour post-FUS together with inflammatory responses. However, it is largely downregulated by 72 hours relative to either control BECs, consistent with reduced inflammation and promotion of angiogenesis. Notch signaling is also upregulated significantly in capillary BECs one hour post-FUS; however, it is shut down by 72 hours post-FUS in capillary BECs, in particular for 750 kPa. Therefore, several signaling pathways critical for angiogenesis and BEC proliferation change dynamically after FUS, but they remain lower in high pressure by 72 hours consistent with more blood vessel damage. Surprisingly, Wnt/β-catenin was not changed at the acute phase for both pressures, compared to the control group. However, canonical Wnt signaling was higher at the acute than the sub-acute (72 hours) phase post-FUS for both pressures and at lower than higher pressures by 72 hours consistent with continuous “EC proliferation” and “angiogenesis” at this time point at 750 kPa. Overall, this analysis demonstrates a “return to normalization” at both pressures by 72 hours since Wnt/β-catenin signaling is reduced once angiogenesis and BBB maturation are complete.^1,2^

Clinical trials currently employ lower frequencies compared to 1.5 MHz used in the current study. Using a lower frequency reduces aberrations caused by the skull and increases the focal volume. While well suited for human studies, lower frequencies pose challenges in small animal studies including the formation of standing waves creating peaks of high pressure associated with damage and heating.^117-119^ Microbubble diameter and the resonance frequency of the transducer will affect how microbubbles oscillate within the vasculature.^19,74^ As clinical trials utilizing FUS-mediated BBB opening for tumor therapy are under way,^23,120^ a rigorous understanding of neurovascular recovery will further aid in enhancing harnessing this technique for therapeutic treatment.

## ONLINE METHODS

### Animals:

All experimental procedures involving animals were approved by the Columbia University Institutional Animal Care and Use Committee (IACUC) and in accordance to the Office of Laboratory Animal Welfare and the Association for Assessment and Accreditation of Laboratory Care regulations. A total of eighteen (N = 18) male and female C57BL6 or *Tg::eGFP-Claudin5* and three (N = 3) male *Tg::eGFP-Claudin5*^*+/−*^*Cav1*^*−/−*^ 12–14 weeks old mice (20-25g) were used for this study. Animals were housed under standard conditions (12 h light/dark cycles, 22°C), were fed a standard rodent chow (3kcal/g; Harlen Laboratories, Indianapolis, IN, USA) and drank distilled water. All mice had access to their diets ad libitum. Animals were group housed and were randomly selected into stable (Pressure = 0.45MPa) or inertial cavitation (Pressure = 0.75MPa) groups.

### Microbubble Preparation:

In-house lipid-shelled microbubbles were manufactured as previously published.^61^ Briefly, the 1,2-distearoyl-sn-glycero-3-phosphocholine (DSPC) and polyethylene Glycol 2000 (PEG2000) were mixed at a 9:1 ratio. Two milligrams of the mixture was dissolved in a 2-ml solution of filtered PBS/glycerol (10% volume)/propylene glycol (10% volume) with a sonicator (Model 1510, Branson Ultrasonics, Danbury, CT, USA) and stored in a 5 ml vial. The remainder of the vial was filled with decafluorobutane (C_4_F_10_) gas. The vial was then activated via mechanical agitation using VialMix^™^ shaker (Lantheus Medical Imaging, N. Billerica, MA) for 45 s. The formed microbubbles were analyzed with a Coulter Counter Multisizer (Beckman Coulter Inc., Fullerton, CA).

### FUS Blood-Brain Barrier Opening:

A single-element 1.5 MHz center frequency focused ultrasound transducer (center frequency: 1.5MHz, focal depth: 60mm, radius: 30mm, axial full width half-maximum intensity: 7.5mm, lateral full-width half-maximum intensity: 1 mm, Imasonic, France) was used for all BBB openings in this study. To measure the beam profile, the transducer was calibrated using a needle hydrophone (HGL-0400, Onda Corp., Sunnyvale, CA) in a tank of deionized, degassed water. The FUS transducer was driven by a function generator (Agilent, Palo Alto, CA, USA) though a 50-dB power amplifier (E&I, Rochester, NY, USA). A pulse-echo transducer (center frequency: 7.5 MHz, focal depth: 60mm, radius 13 mm; Olympus NDT, Waltham, MA) was confocally aligned with the FUS transducer to monitor cavitation events. The pulse-echo transducer was driven by a pulse-receiver (Olympus NDT, Waltham, MA) connected to a digitizer (Gage Applied technologies, Inc., Lachine, QC, Canada) for data acquisition. During passive cavitation detection (PCD) acquisition the pulse-receiver operated in “receive mode” and served as an amplifier during PCD acquisition. The transducer setup was then mounted onto a three-dimensional positioning system (Velmex Inc., Lachine, QC, Canada) for accurate targeting.

A bolus of 1μL/g of body mass of polydisperse microbubbles diluted in (8X10^8^/mL, mean diameter: 4.5 μm) with 100μL was intravenously injected immediately preceding the sonication. For experiments involving *ex vivo* visualization of biocytin diffusion, mice survived for 1 hr were co-injected with the fluorescent tracer biocytin-tetramethylrhodamine (870 Da, Life Technologies). The caudate putamen is a biologically relevant structure for diseases such as Alzheimer’s and Parkinson’s, and a structure that has been previously targeted for drug delivery^62^. Targeting of the caudate putamen was achieved by first locating the lambda structure though an intact dilapidated scalp and skull as previously published ^63^. Briefly, a water cone filled with deionized (DI), and degassed water was fixed to the FUS transducer, which was submerged in a water bath of DI and degassed water, and finally coupled to the shaved scalp using degassed ultrasound gel. A metallic wire with orthogonal parts was placed above the lambda structure and a C-mode ultrasound was performed to locate the cross and below it, the lambda structure. The FUS transducer was then moved via the positioner to the following coordinates from lambda: AP 4.5 mm, ML 2.5 mm and DV −3.5 mm for caudate targeting and a single sonication was performed. The FUS parameters used were as follows: frequency 1.5 MHz, peak-rarefactional pressure 0.45 MPa or 0.75 MPa, pulse length 10 ms, pulse repetition frequency 10 Hz, and for a duration of 120 s.

### Magnetic Resonance Imaging:

One hour following the ultrasound procedure, all animals underwent scanning with the 9.4T MRI system (Bruker Medical, Boston, MA). The mice were placed in a birdcage coil (diameter 35 mm), while being anesthetized with 1–2% isoflurane and respiration rate was monitored throughout the imaging sessions. MR images were acquired using a contrast-enhanced T1-weighted 2D FLASH sequence (TR/TE 230/3.3 ms, flip angle: 70°, number of excitations: 6, field of view: 25.6 mm × 25.6 mm, resolution 100 μm x 100 μm x 400 μm), 45 min following the intraperitoneal bolus injection of 0.3 ml gadodiamide (GD-DTPA) (OmniscanTM, GE Healthcare, Princeton, NJ). As previously reported, gadodiamide provides spatial information of the BBB opening, as it will diffuse into the brain where the BBB disrupted, enhancing MR signal temporarily and enabling *in vivo* visualization of the BBB opening volume.^64^ Mice survived for 72 hours, underwent an additional scan of the same sequence, 71 hrs following FUS.

MRI BBB Opening Volume Quantification: Contrast-enhanced T1-weighted MRI images were processed in MATLAB (2019a Mathworks, Natick, MA,USA). Analysis was performed on 15 consecutive coronal images that were manually segmented to remove the skull from the brain. The volume of BBBO, or BBB disruption is defined for the purposes of this study as the volume of the brain into which Gd contrast agent is able to diffuse and be visualized on T1-weighted MRI. This affected volume will be the focus of subsequent histological and transcriptomic analyses. Images were thresholded to generate a binary mask based on the mean plus two standard deviations pixel intensity of an ROI chosen on the contralateral hemisphere. The area of each mask was calculated and summed across all 15 slices to give the final BBB opening volume.

### Immunofluorescence Staining and Imaging:

For the biocytin-TMR quantification, mice survived for 72 hours, were injected with biocytin-TMR one hour prior to sacrifice. Either 1 or 72 hours post-sonication, mice were transcardially perfused with 30 mL PBS followed by 60 mL 4% paraformaldehyde. The skull was removed and the brain was soaked in paraformaldehyde for six hours, transferred to PBS overnight, cryoprotected in 30% sucrose for 48 hours, and then frozen on dry ice. Brains were coronally sectioned at 25μm throughout the caudate putamen region. A series of 4–6 sections within the focus (1mm) centered at Bregma 0.3 mm were serially selected for each mouse for staining. Tissues were stained for eGFP (1:1000; Sigma-Aldrich, MO), Glut-1 (1:1000; EMD Millipore, MA), DyLight 649 Labeled Griffonia (Bandeiraea) simplicifolia lectin I (1:250; Vector Laboratories, CA), Streptavidin-Alexa594 (1:1000; ThermoFisher) was used to visualize biocytin-TMR distribution in tissues,^39^ Iba-1 (1:100; Abcam), CD68 (1:100; Abcam), Fibrinogen (1:500; EMD Millipore, MA), and GFAP (1:1000, Abcam). Whole brain images were captured with an upright Zeiss AxioImager. Confocal images for analysis of tight junction morphology were captured on a Zeiss LSM700 confocal microscope. For each section, 4–6 images were captured on both the contralateral and ipsilateral hemisphere exclusively within the caudate putamen. Images on the ipsilateral hemisphere were acquired near sites of BBB opening, determined by the presence of Biocytin-TMR. Images were minimally processed with Fiji/ImageJ software (NIH) to enhance brightness and contrast.

### In Situ Hybridization

Fluorescent *in situ* hybridization combined with IF of fresh-frozen sections using antisense mRNA probes for *Egfl7* and *Angpt2*transcripts and Glut-1 (vessel marker) were performed as previously described.^55^ The sections were imaged at 20x using a Zeiss LSM900 confocal microscope.

### Acoustic Signal Analysis

For each FUS pulse transmitted, microbubble cavitation was acquired by a single element PCD, transferred to a digitizer (GAGE Applied Technologies Inc, Lachine QC, Canada), and processed in MATLAB (2017a Mathworks, Natick, MA, USA). Each PCD pulse was transformed into a power spectrum using a fast Fourier transform (fs = 50 MHz) and the resulting energy spectral density was bandpass filtered (3MHz - 9 MHz). The peak value within a 20 kHz bandwidth around each harmonic (nf_c_, n = 1, 2…6, f_c_=1.5 MHz) and ultraharmonic (nf_c_/2, n = 3, 5, 7, 9) was found. The cavitation dose is computed as the root mean square (rms) of the spectral amplitude for each time point. SCDu and SCDh are given by the CD of their respective bins. The inertial cavitation dose (ICD) is defined as any CD energy not contributing to SCDh or SCDu. The cumulative cavitation dose is given integrating the amplitude over the entire sonication duration. The cumulative cavitation emissions from microbubbles were normalized to baseline cavitation dose measured prior to IV injection of bubbles.

### Single-Cell RNA Sequencing Preparation

At either 1 or 72 hr. post-FUS, eGFP-Cldn5 mice were deeply anesthetized with 4% isoflurane and perfused for 4 minutes with 4°C, sterile PBS. The brain was dissected into Earl's balanced salt solution (EBSS) and dissociated into a single cell suspension using an established protocol.^55^ The single-cell suspension was resuspended in 200 μl of CD16/CD32 Fc block (BD Biosciences, Cat. # 553141; 1/200 in FACS buffer) and incubated at room temperature for 15 minutes. Samples were washed with 2 ml of FACS buffer, and 100 μl were kept aside for unstained and fluorescence minus one (FMO) controls. The cells were resuspended in 200 μl of anti-CD31-APC (Biolegend, Cat. # 102410; 1/200 in FACS buffer) antibody to label endothelial cells (ECs) and incubated on ice in the dark for 30–60 mins. Following antibody labeling, the samples were washed twice with 2 ml of FACS buffer and resuspended in 400 μl of propidium iodide (Thermofisher, Cat. # P1304MP; 1/10,000 in FACS buffer). Gates for the surface stain were set using the unstained and FMO controls. The double, eGFP-Cldn5 and CD31-APC positive ECs were sorted out using the BD FACS Aria Flow Cytometer (Columbia Stem Cell Initiative Flow Cytometry Core) and processed for scRNAseq.

## QUANTIFICATION AND STATISTICAL ANALYSIS

### BBB Leakage and Vessel Size Quantification

Whole brain slices were used to quantify the intensity of the fluorescent tracer Biocytin-TMR and the vasculature by immunofluorescence staining for GLUT-1. Leaky vessels were defined as segmented vessels that colocalized with areas of leakage determined by a fixed threshold. Biocytin-TMR leakage was quantified with a custom algorithm in MATLAB (R2017a, Mathworks, Inc., Natick, MA, USA). For quantification of fluorescence intensity, an ROI of the entire sonicated hemisphere containing the caudate putamen and the other brain structures affected by the FUS focus, such as the somatosensory cortex, was selected for each section in the ipsilateral and contralateral side and fluorescence intensity was calculated by subtracting the selected area times the mean fluorescence of the background from the sum of the pixel values of the ROI. Vessels were identified by GLUT-1 staining, and a custom script written in MATLAB (R2017a, Mathworks, Inc., Natick, MA, USA) segmented vessels and measured the diameter. Leaky vessels were defined as segmented vessels that colocalized with areas of biocytin-TMR leakage determined by a fixed threshold. For quantification of the percentage of leaky vessels for small and large caliber vessels, the number of leaky vessel for either small or large vessels was divided by the total number of small or large vessels in the ROI. For each identified vessel the area of biocytin-TMR leakage above a fixed threshold and bioctin-TMR intensity was computed within an ROI centered around the vessel.

### TJ Quantification:

Maximum intensity projections were created in order to quantify TJ abnormalities. The number of gaps was quantified for each TJ junction strand in leaking vessels on the ipsilateral side, and control vessels on the contralateral side. Vessel boundaries were determined by GLUT-1 staining. Arterioles, venules, and capillaries were identified based on 1) diameter of the vessel to separate capillaries from arteiroles and venules and 2) tight junction structure to distinguish between the three vessel types. Colocalization with leakage of the fluorescent tracer Biocytin-TMR (870 Da) with venules, identified by their TJ morphology, were not found, therefore analysis of venules was excluded from this analysis. Gaps and protrusions are defined as described.^5,39^ Only TJ strands contained within vessel boundaries were included in the analysis. Tight junction gap length was measured with Fiji/Image J software (NIH) using maximum intensity projection images from Tg::eGFP-Claudin5 mice one hour after FUS. Histograms of the length of tight junction disruption pooled across mice and vessel type demonstrated a bimodal distribution, and a cutoff threshold of 2.5 μm was chosen. Data was sorted based on gap lengths from 0.4–2.5μm and gaps greater than 2.5 μm.

### Immunofluorescence Area Quantification

Maximum intensity projections were created and pixels were thresholded determined by the mean plus two standard deviations of a manually selected ROI containing background noise. For a given immunofluorescent stain, all images underwent the same processing pipeline regardless of hemisphere, pressure, or time point.

### Single-Cell RNA Sequencing Analysis:

For single-cell RNA sequencing (scRNAseq), the 10x Genomics Chromium platform was used to generate low-depth data (~ 2000 genes/cell), which was processed using the Cell Ranger analysis pipeline to align reads and generate feature-barcode matrices. The Seurat R package^56^ was implemented to read the output of the Cell Ranger pipeline and merge cells from all the samples (untreated controls, high and low FUS pressures, 1 hr. and 72 hr. timepoints) into a single R object. The standard pre-processing workflow for scRNAseq data was carried out in Seurat: cells were filtered based on quality control metrics (number of unique genes detected in each cell > 200, total number of molecules detected within a cell > 1,000 and < 50,000, percentage of reads within a cell that map to the mitochondrial genome < 20, etc.), the data was normalized and scaled, and highly variable features were detected. Next, linear dimensionality reduction (PCA) was performed on the scaled data using the previously determined highly variable features, an alternative heuristic method ('Elbow plot') was implemented to determine the 'dimensionality' of the dataset, and cells were clustered by applying the weighted shared nearest-neighbor graph-based clustering method.^66^ Finally, the PCA dimensions were further reduced into two-dimensional space using the non-linear dimensional reduction technique, t-distributed stochastic neighbor embedding (t-SNE), to visualize and explore the scRNAseq dataset and each of its unique clusters. To ensure the purity of endothelial cells in subsequent analysis, all sequenced cells were clustered and the expression levels of canonical cell-type markers Cldn5, Ptprc, Gfap, Pdgfrb, Map2, and Acta2 were evaluated across the clusters. A cluster of contaminating pericytes was identified and excluded based on high expression of Pdgfrb and Acta2. The remaining, pure endothelial cells were then segmented out and clustered again. The expression of vessel subtype markers such as arterial (Bmx, Efnb2, Hey1, and Alpl), capillary (Ivns1abp, Slc1a1, Mfsd2a) and vein (Nr2f2, Len2, and Prcp) were used to identified arterial, capillary and vein endothelial cells as shown in Fig. 2C.

### Differential Gene Expression Analysis

The Seurat “FindMarkers” function with the default Wilcoxon Rank Sum test was implemented to compile the lists of differentially expressed genes, by comparing expression profiles of FUS-BBBO EC clusters to control EC clusters at 450 and 750 kPa, at both acute (1 hr.) and long-term (72 hr.) timepoints.

### Gene Set Enrichment Analysis (GSEA):

Differential gene expression (DE) analysis was performed to compare gene expression variation between experimental groups^67^ and highlight which clusters have further heterogeneity associated with each condition. Following DE analysis, a score was calculated for each gene using the formula: Gene score = −log10(pval) x sign (log2fc). Up- and downregulated genes had a positive and negative score, respectively. The numeric value of the gene score was inversely correlated with the degree of significance, that is, genes that had the highest statistically significant difference in expression and the lowest p-values had the highest score, while non-significant genes with high p-values had a correspondingly low gene score. This score was used to rank each gene in DE gene list. Gene sets relevant to pathways, processes and cell types of interest (blood-brain barrier, VEGF signaling, canonical Wnt/β-catenin signaling, non-canonical Wnt signaling, TGF-β signaling, extracellular matrix, cell-cell adhesion, transporters, Notch signaling, angiogenesis, endothelial cell proliferation, endothelial cell migration, apoptosis, antigen processing and presentation, inflammation, endothelial-to-mesenchymal transition, tip cells) were compiled as part of the overall gene set enrichment analysis, using the GO, KEGG, DAVID and GSEA databases as published previously.^68-70^ The ranked list of DE genes, along with the compiled gene sets, were loaded onto GSEA 4.1.0 software, and run through ‘GseaPreranked’ with the following settings: Number of permutations = 1,000; Collapse/Remap to gene symbols = No_collapse; Max size: exclude larger sets = 1,500; all other settings were left at ‘default’. Gene sets with FDR q value ≤ 0.3 were deemed significantly enriched and are included in the GSEA graphs.

### Statistical Analysis:

Data presented as bar graphs indicate mean ± standard deviation (SD). Dots in bar graph represent individual values per mouse. Pairwise comparisons were calculated with unpaired or paired two-tailed t tests. Comparisons of one or more groups were performed using a 2-way ANOVA and p-values were adjusted based on the Holm Sidak post hoc correction. Statistical analyses were performed in GraphPad Prism (GraphPad Software, San Diego, CA, USA). Statistical details of experiments can be found in figure legends. Sample size was not predetermined using power analysis. Statistical differences between groups in Fig. 8 are determined by Wilcoxon rank sum test comparing each group to the untreated control. Significance levels are indicated as follows: *p < 0.05; **p < 0.01; ***p < 0.001; ****p < 0.0001.
